# GiA Roots: software for the high throughput analysis of plant root system architecture

**DOI:** 10.1186/1471-2229-12-116

**Published:** 2012-07-26

**Authors:** Taras Galkovskyi, Yuriy Mileyko, Alexander Bucksch, Brad Moore, Olga Symonova, Charles A Price, Christopher N Topp, Anjali S Iyer-Pascuzzi, Paul R Zurek, Suqin Fang, John Harer, Philip N Benfey, Joshua S Weitz

**Affiliations:** 1 Department of Mathematics, Duke University, Durham, NC, USA; 2 School of Biology, Georgia Institute of Technology, Atlanta, GA, USA; 3 School of Interactive Computing, Georgia Institute of Technology, Atlanta, GA, USA; 4 Department of Biology, Duke University, Durham, NC, USA; 5, Institute of Science and Technology, Vienna, Austria; 6 Department of Plant Biology, University of Western Australia, Perth, Australia; 7 Duke Center for Systems Biology, Duke University, Durham, NC, USA; 8 Root Biology Center, South China Agricultural University, Guangzhou, China; 9 School of Physics, Georgia Institute of Technology, Atlanta, GA, USA

## Abstract

**Background:**

Characterizing root system architecture (RSA) is essential to understanding the development and function of vascular plants. Identifying RSA-associated genes also represents an underexplored opportunity for crop improvement. Software tools are needed to accelerate the pace at which quantitative traits of RSA are estimated from images of root networks.

**Results:**

We have developed GiA Roots (General Image Analysis of Roots), a semi-automated software tool designed specifically for the high-throughput analysis of root system images. GiA Roots includes user-assisted algorithms to distinguish root from background and a fully automated pipeline that extracts dozens of root system phenotypes. Quantitative information on each phenotype, along with intermediate steps for full reproducibility, is returned to the end-user for downstream analysis. GiA Roots has a GUI front end and a command-line interface for interweaving the software into large-scale workflows. GiA Roots can also be extended to estimate novel phenotypes specified by the end-user.

**Conclusions:**

We demonstrate the use of GiA Roots on a set of 2393 images of rice roots representing 12 genotypes from the species *Oryza sativa*. We validate trait measurements against prior analyses of this image set that demonstrated that RSA traits are likely heritable and associated with genotypic differences. Moreover, we demonstrate that GiA Roots is extensible and an end-user can add functionality so that GiA Roots can estimate novel RSA traits. In summary, we show that the software can function as an efficient tool as part of a workflow to move from large numbers of root images to downstream analysis.

## Background

Plant roots are essential to the structure and function of plants. They provide access to belowground water and nutrients [1-3], facilitate anchorage of plants in soils [4,5], mediate chemical defense belowground [6,7], and serve as sites of important symbioses with microbiota [8,9]. However, comparatively little is known about the architecture of plant root systems and their relationship to overall plant function given the relative inaccessibility of belowground tissue [10]. Recently, standardized field procedures have been developed for quantifying RSA using excavated and washed roots [11]. In addition, a number of non-destructive approaches to imaging and quantifying RSA have been developed. The approaches include X-ray computed tomography [12-15], nuclear magnetic resonance (NMR) microscopy [16], magnetic resonance imaging [17,18], laser scanning [19], as well as a growing number of imaging methods using plants grown in transparent media [20-25]. These methods are part of a rapidly growing field of “plant phenomics” whose objective is to link plant genotypes to plant phenotypes [26-29], particularly in the service of plant biotechnology and crop improvement [30,31].

Analyzing large numbers of plant root system images requires the use of software tools specifically designed for the high-throughput estimation of RSA traits. A number of different tools are available for the analysis of RSA. For example, some programs are specialized for the analysis of images from a specific apparatus, e.g., images from minirhizotrons [32-35]. Next, general tools are available for in-depth analysis of individual monocot root systems regardless of apparatus. These tools rely on significant user input for processing although they can be used in a batch mode [22,36,37]. Similarly, general purpose image processing programs such as ImageJ may be flexible enough to perform many specialized tasks [38]. In practice, end-users often utilize point-and-click approaches that are not scaleable to large numbers of edges within a root system (for an exception, see the recently released software “SmartRoot” which is designed for semi-automated analysis of the hierarchical structure of root systems [39]). Finally, software is also available that can accurately characterize the simpler dicot root system of *Arabidopsis*[23,24], in a high-throughput fashion [40], but that is not yet suitable for studying the intricate monocot root systems of rice and maize.

GiA Roots is a software tool that estimates RSA traits from a large number of root system images. The main distinguishing characteristics of GiA Roots are that it is both specifically designed for high-throughput analysis and it is extensible. A typical user begins by interactively setting parameters to enable GiA Roots to identify roots from the background, i.e., segmenting the image. Next, the user selects traits of interest for measurement. Finally, the user instructs GiA Roots to automatically estimate traits for images. The GiA Roots pipeline of steps can be executed from the GUI or command line tool as part of a fully automated workflow. A technical user can add new traits to the GiA Roots pipeline using the application programming interface of GiA Roots (GiA-API).

In this manuscript, we describe the implementation of the software, including the main computational steps. We discuss the use of GiA Roots, including validating its measurements via estimation of 19 RSA traits on a large-scale data set of 2393 images from rice root systems. We demonstrate the segmentation capabilities of GiA Roots on root system images. We also demonstrate how GiA Roots can be extended to include a novel trait presented in a recent RSA analysis of 3D reconstructions of root networks [22] but not contained in our earlier study of 2D images of root networks [21]. Finally, we discuss the limitations of the software and plans for further development.

## Implementation

**Figure 1:**
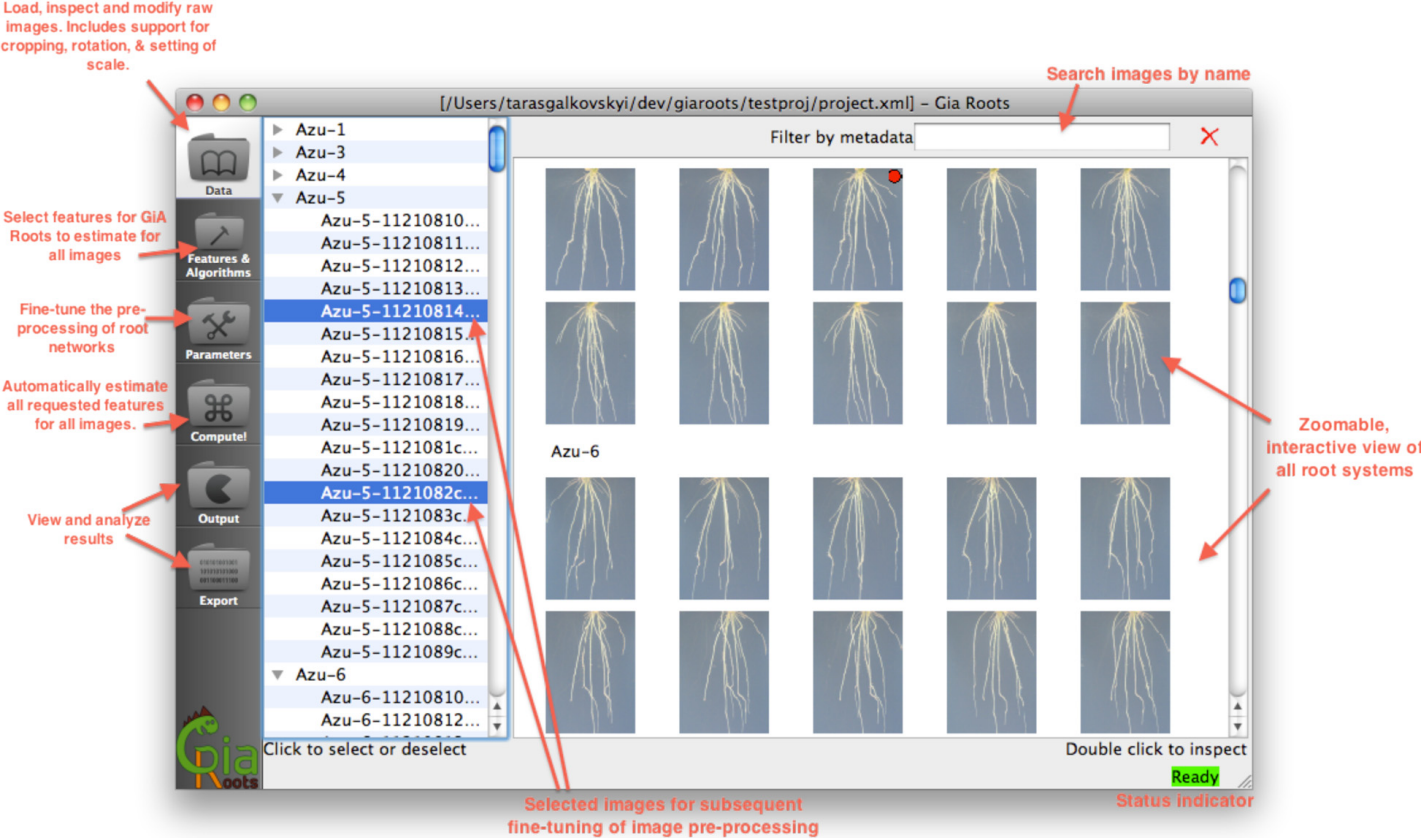
**Annotated snapshot of the GiA Roots GUI.** The GiA Roots GUI is a standalone application that provides a user interface to manage input images, define a processing pipeline and manage output. It features a main window with several task windows that can be accessed through the left sidebar. Sequential access to each window page accomplishes major tasks: managing data; selecting traits to measure; tweaking parameters; performing processing; reviewing the results. This linear design helps users to keep track of progress and proceed intuitively with processing of the data. Main computational steps are described in greater detail in Implementation.

GiA Roots is implemented in C++ and is comprised of two applications: a GUI and a command-line tool. The core component of GiA Roots is a trait estimation pipeline. The trait estimation pipeline includes: an optional user-assisted processing of images, scale calibration, trait selection, image segmentation (*i.e.* the separation of root pixels from background), trait measurement, and output. The GUI and command-line tool can be used together. The GUI workflow provides an interactive means to utilize the trait estimation pipeline of the command line tool (see Figure 1). Hence, the GiA Roots GUI helps users fine-tune the performance of GiA Roots’s image processing steps. The command-line tool facilitates the high throughput processing of large data sets.

### Trait estimation workflow

#### Importing imaging data

A user starts GiA Roots by specifying a set of root images to load. Two different import modes allow users to import either (i) sets of images from one folder; or (ii) the whole hierarchy of folders that contain images. Images are automatically organized into stacks grouped by the folder they were imported from. It is possible to browse all imported images, or filter only a subset of images (i.e., “image-stack”), based on certain search criteria.

#### Image pre-processing and scale calibration

GiA Roots can apply optional pre-processing transformations to images. The transformations are cropping, rotation and scale calibration. Transformations can be applied on a per-image, per-image stack and an all-images basis. Cropping and rotation are standard image transformations. Scale calibration allows users to set the scale in terms of pixels/cm. This functionality is accessible from the detailed view of any image in the project. Users can either draw a line that corresponds to a specified distance of the scale bar (in cm) or enter the scale value. The scale value can be applied to the current image, current image stack, or all images in the project. GiA Roots retains dimensional information when processing, so that trait estimates will be reported in the proper units, such as *cm*, *cm*^2^ or *cm*^3^. When a scale is not specified for an image, its traits will not be scaled and will be presented in units of pixels.

#### Trait selection

Users can select traits to be estimated from a list of 19 established RSA traits. Illustrated descriptions are provided in the GUI for each trait to detail the relationship between the trait and the structure of the plant. The traits included in this distribution of GiA Roots are based upon two sources [21,22], and include the following: aspect ratio, average width of roots, bushiness, convex area, network depth, network length distribution, major ellipse axis, maximum number of roots, maximum width of root system, median number of roots, minimum ellipse axis, network area, network length, network solidity, network surface area, network volume, network width to depth ratio, perimeter, and specific root length. Specifications for these traits can be found in Table 1 and more information can be found in [21,22]. Additionally, each intermediate processing step can also be selected for output.

**Table 1:** Traits description

		
**Trait**	**Units**	**Description**
Bushiness (Bush)	*n*/*n*	The ratio of the maximum to the median number of roots
Convex area (ConvA)	*cm*^2^	The area of the convex hull that encompasses the root.
Network depth (Ndepth)	*cm*	The number of pixels in the vertical direction from the upper-most network pixel to the lower-most network pixel.
Network Length Distribution (Ldist)	*n*/*n*	The fraction of network pixels found in the lower 2/3 of the network. The lower 2/3 of the network is defined based on the network depth.
Major axis (MajA)	*cm*	The length of the major axis of the best fitting ellipse to the network
Network Width (Nwidth)	*cm*	The number of pixels in the horizontal direction from the left-most network pixel to the right-most network pixel. Only pixels lying in the same row are considered.
Maximum number of roots (MaxR)	*n*	After sorting the number of roots crossing a horizontal line from smallest to largest, the maximum number is considered to be the 84th-percentile value (one standard deviation).
Average root width (Width)	*cm*	The mean value of the root width estimation computed for all pixels of the medial axis of the entire root system. This trait corresponds to diameter of a root.
Median number of roots (MedR)	*n*	The result of a vertical line sweep in which the number of roots that crossed a horizontal line was estimated, and then the median of all values for the extent of the network was calculated.
Minor axis (MinA)	*cm*	The length of the minor axis of the best fitting ellipse to the network
Network area (NwA)	*cm*^2^	The number of network pixels in the image.
Perimeter (Perim)	*cm*	The total number of network pixels connected to a background pixel (using a 8-nearest neighbor neighborhood).
Aspect ratio (AspR)	*cm*/*cm*	The ratio of the minor to the major axis of best fitting ellipse
Network solidity	*cm*^2^/*cm*^2^	The total network area divided by the network convex area.
Specific root length (SRL)	*cm*/*cm*^3^	Total network length divided by network volume. Volume is estimated as the sum of cross sectional areas for all pixels of the medial axis of the root system. The total root length is the number of pixels in the medial axis of the root system.
Network Surface Area (Nsurf)	*cm*^2^	The sum of the local surface area at each pixel of the network skeleton, as approximated by a tubular shape whose radius is estimated from the image.
Network length (Nlen)	*cm*	The total number of pixels in the network skeleton.
Network volume (Nvol)	*cm*^3^	The sum of the local volume at each pixel of the network skeleton, as approximated by a tubular shape whose radius is estimated from the image.
Network width to depth ratio	*cm*/*cm*	The value of network width divided by the value of network depth.

#### Image processing via segmentation – extracting roots from background

Trait estimation in GiA Roots is done on “segmented” images, in which root pixels are separated from background pixels. Segmentation can be applied on a per-image, per-image stack and an all-images basis. GiA Roots offers three thresholding methods to segment the image into foreground (root) and background. The methods are: global thresholding, adaptive thresholding, and double adaptive thresholding. Global thresholding identifies all roots above a critical pixel intensity as part of the root and all other pixels as background. Adaptive thresholding divides the image into small square regions, calculates the mean pixel intensity in each region, and then decides whether a pixel is part of the root based on whether its intensity exceeds that of the mean intensity in each region by a user-defined offset. Finally, double adaptive thresholding evaluates whether or not a given pixel is part of the root by growing a square centered around the pixel and checking if mean intensity in the square decreases rapidly before the square reaches a critical size. This decrease is common in cases where a group of root pixels are brighter than the background. In all cases, we also provide an additional step in which small connected groups of pixels are removed from the segmented image.

Each thresholding algorithm has default parameter values that facilitate segmentation of foreground and background (see Table 2 and additional information in the GiA Roots manual). Multiple algorithms are provided given the commonly noted inadequacy of any single thresholding algorithm to serve as a universal best choice depending on the application [41,42]. For non-technical users, GiA Roots provides an interactive means to facilitate rapid review and improvement of segmentation. Sliders are available in the GUI in which users can modify thresholding parameters and view the result of segmentation immediately. Hence, users can tweak parameters until they are satisfied with the expected output. Users can save thresholding configurations for later use in the GUI or in the command line tool. GiA Roots saves all configurations along with intermediate images and intermediate numerical results to a chosen folder. GiA Roots therefore enables reproducibility of results with known configuration sets.

**Table 2:** Segmentation parameters


**Thresholding Algorithm**	**Parameter**	**Description**
Global thresholding	*Threshold value*	Threshold value of pixel intensity to be considered as part of the root.
Adaptive thresholding	*Block size*	The size of square regions to measure the average local pixel intensity.
…	*Mean shift*	Threshold offset that a focal pixel intensity must exceed relative to the pixel intensity averaged over a local region for it to be considered as part of the root.
Double adaptive thresholding	*Neighborhood size*	Critical size of a growing square centered on a pixel for which average pixel intensity is calculated.
…	*Bound drop value*	Threshold decrease in average pixel intensity in square neighborhoods of increasing size that must be reached for a pixel to be considered as part of the root.
All methods	*Ignore con comp of size less*	All connected components with fewer than this many pixels are removed.

#### Trait estimation

After thresholding parameters are specified, the user directs GiA Roots to compute traits from a set of specified images. GiA Roots computes traits directly from the image mask or by computing properties of the skeleton of the image mask (see Table 1). We have chosen the medial axis transformation as a skeletonization method; this transformation is equivalent to a morphological thinning on a binary mask that preserves the topology of the original image [43]. Different skeletonization algorithms can be implemented into GiA Roots via the GiA-API. The GUI workflow follows the processing pipeline of the GiA Roots command line tool. Hence, there is no user involvement in trait computation. In the GUI, partial results of trait estimation can be accessed while computing the RSA traits of all selected images. Preliminary checks on image stacks can be used to test if a given set of configuration parameters yields satisfactory output. In practice, we recommend that users save configuration files in the GUI when analyzing a large number of images. Subsequently, a user may open a saved configuration file which speeds up the workflow prior to executing the computation. Configuration files can also be utilized as part of the command line user interface.

#### Output

There are two methods to export measurements obtained by GiA Roots. First, numerical measurements can be exported in the form of a comma-separated value (.csv) file suitable for analysis in spreadsheet or other statistical analysis package. Second, image data of all processing steps can be exported to verify results. For example, segmented root images or root image skeletons can be selected for export. All processing intermediates can also be selected for export to verify the performance of GiA Roots.

### Automation and integration

GiA Roots can handle small to large data sets, from a single image to many thousands of images. The GUI is particularly well-suited for identifying processing parameters that facilitate estimation of root network traits. Large-scale analysis can also be conducted using the GUI. The command-line tool provides an automated means for processing large numbers of images given configuration parameters (e.g., determined using the GUI). Specifications are contained in an XML file (see Manual). The XML file allows for control of each step of the processing pipeline from algorithm parameters to selected traits to compute. Practically, this enables the GiA Roots pipeline to be integrated with 3rd party scripts or applications. GiA Roots also exposes the intermediate output of all processing steps. This allows users to redirect parts of the GiA Roots pipeline into their workflows. For example, a user could use the output of the segmentation step as input into a 3D reconstruction application. It is important to note that both the GUI and command-line tool output a project XML file that contains enough information to reproduce any output or intermediate outputs.

In practice, the integration of GUI and command-line tool works as follows. Consider a scenario in which users take images of root systems using the same imaging platform and setup. First, the user runs the GiA Roots GUI to fine-tune the image processing steps for a sample of images and determines which RSA traits to measure. Once confirmed, these settings can be exported from the GUI as an XML configuration file. RSA traits of the entire image set can be estimated using the GUI or via the command line tool using the saved XML configuration file. When new data is available, the user executes the command-line tool with the exported configuration file and a job file describing the new incoming data (see Manual for more details). The execution of the command-line tool will be non-interactive (except for displaying a progress log). The command line tool produces the same results as the GUI. Furthermore, since the tool is non-interactive, multiple instances of it can be run on multiple servers. Each instance can also be configured to exploit the parallel architecture of a single machine using multi-threading.

### Extending GiA Roots

**Figure 2:**
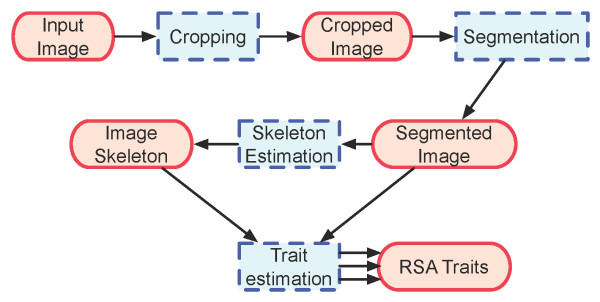
**GiA Roots processing chart.** Data types are enclosed in ellipses, interactions are enclosed in rectangles. Interactions are realized by plugins, and have several variants. They can also be configured.

Technical users may be interested in extending GiA Roots so that it can estimate novel traits. Such extensions are made possible by the general processing scheme in GiA Roots (see Figure 2). In GiA Roots, there are a pre-defined set of data types (e.g., raw image, cropped images, transformed image, etc.) and a set of transformations from one data type into another. Each of the transformations is realized as a software plugin. However, multiple algorithms can be associated with each transformation. For example, a “segmentation” plugin is necessary in order to transform a cropped image into a segmented image. GiA Roots is pre-bundled with three different segmentation plugins: global thresholding, adaptive thresholding, and double adaptive thresholding (see prior text and GiA Roots manual for more details). GiA Roots enables users to specify which plugin to utilize in either the GUI or the command-line tool. Further, users can specify parameters specific to each plug-in to optimize the performance for their image cases.

Importantly, all transformations are independent of one another. This independence is retained by a software framework design in which data types are the only ways that plugins can communicate. Hence, developers can add a plugin to function as an alternative algorithm to a pre-existing transformation (e.g., segmentation) or trait estimation step (e.g., calculating the total length of the network). Of course, the outputs of algorithms do interact with each other, so modification of a segmentation algorithm that leads to a different segmented image may lead to a different image skeleton. In developing a new feature, developers must ensure that their plug-ins fit into a specific location in a processing pipeline and strictly comply with uniqueness requirements for both the input and output data type. Developers must specify the dependency of the plugin based on the data types it requires and the data types it outputs. Description of the APIs for adding new features can be found in the manual (see project homepage).

## Results and discussion

### GiA Roots can estimate RSA traits for thousands (and more) root images

**Figure 3:**
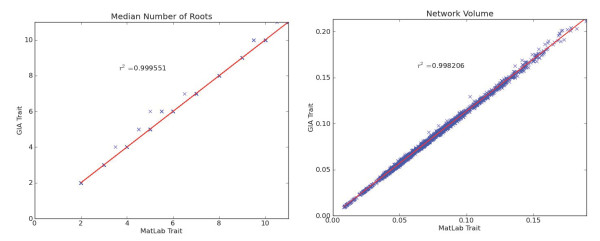
**Comparison of GiA Roots estimation of trait values against a prior benchmark.** The two plots represent a comparison of GiA Roots estimation of trait values compared against a previously validated set of algorithms [21]. Each point represents a trait estimate from one of 2393 images. Note that the median number of roots is defined to be an integer so nearly all comparisons in the left panel exactly coincide, but when plotted they appear to give rise to a ‘gridded’ pattern. The *R*^2^values confirm the strong correspondence of the two implementations of the same trait.

Here, we demonstrate that the output of trait estimation algorithms in GiA Roots is equivalent to a previously validated set of algorithms for estimating RSA traits [21]. In the previous study [21], RSA traits of a manufactured “root” wire model were estimated using a MATLAB-based processing pipeline. The estimates were shown to be in close agreement with hand measurements (0.5*%* to 6.7*%*differences) [21]. GiA Roots leverages the OpenCV libary to estimate many of the same traits (in addition to novel traits) as estimated in the prior MATLAB-based pipeline. Hence, we anticipate that estimates of traits in GiA Roots should coincide with prior estimates. We compared GiA Roots against the output of the prior MATLAB-based processing pipeline given a set of 2393 segmented rice root images from 12 genotypes (see http://www.rootnet.biology.gatech.edu/data/PlantPhysData.zip for the complete set of images). In Figure 3 we show the correspondence between the MATLAB-based output (x-axis) and the GiA Roots-based output (y-axis) for two traits where each point in a panel denotes a given image. The two traits we include are median number of roots and network volume. All comparisons can be found in Additional file 1. The results correlated as expected with an *R*^2^≈1.0. Note that small differences in trait estimates can arise due to implementation differences in the medial axis transformation. There are two caveats involving refinement of trait definitions between the study of [21] and the current study. Previously, network length distribution was defined as the ratio of “the total root length in the upper one-third of the root depth to the total root length located in the lower two-thirds of the root depth” [21]. Whereas here, we re-define this trait as the fraction of network pixels found in the lower 2/3 of the network, where the lower 2/3 of the network is defined based on the network depth (see Table 1 for trait descriptors). In addition, we previously reported the thickness of roots as the average root radius [21], whereas now we define it as the average root diameter (hence there is a factor of 2 difference which we correct in evaluating the correspondence). Note that GiA Roots also includes three novel traits not contained in the previous study [21], these are properties of the best-fit ellipse: (i) minor ellipse axis length; (ii) major ellipse axis length; and (iii) ellipse axis ratio. We validated our implementation of these features in GiA Roots against a MATLAB implementation. All ellipse-related traits are highly correlated with *R*^2^≈1 as before. The comparisons are included in Additional file 1. Processing of the dataset from [21] took 2 hours and 28 minutes for 2393 images on a MacBook Pro, 2.2 Ghz Intel i7, 4GB DDR3 RAM, with Mac OSX 10.6.8. Runtime performance of GiA Roots is comparable to the MATLAB implementation. However, the ability to interactively adjust parameters is available in GiA Roots and is not available in the MATLAB implementation.

### GiA Roots identifies roots from noisy images

**Figure 4:**
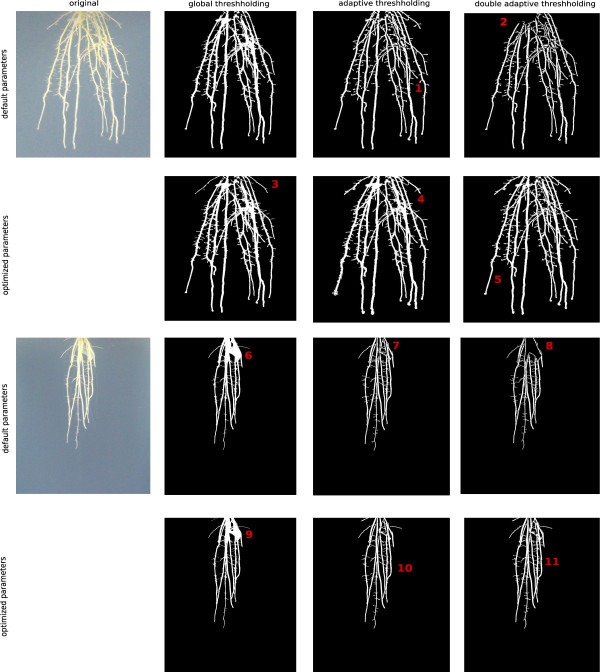
**Comparison of the three available thresholding algorithms in GiA Roots.** The three algorithms are global thresholding (GT), adaptive thresholding (AT), and double adaptive thresholding (DAT). Each algorithm is demonstrated on two relevant examples with default parameter settings and manually optimized parameter settings. Annotations 1-11 point at significant differences which are detailed in the text.

GiA Roots utilizes a thresholding algorithm to segment foreground (roots) from background. The three algorithms available in GiA Roots are global thresholding (GT), adaptive thresholding (AT), and double-adaptive thresholding (DAT). As stated before, the choice of the thresholding algorithm is highly dependent on the provided image quality [41,42]. Here, we visually compare the thresholding algorithms provided in GiA Roots with two root images (see Figure 4). The two images are selected from the publicly available data set in [21] and correspond to individuals on the 14th day after planting from the rice genotypes: Jefferson (top) and Teqing (bottom). We compare the default and manually optimized parameters for each of the two images as summarized in Table 3. The annotations in Figure 4 illustrate differences between the default parameters and those improved via user-assisted optimization.

**Table 3:** Parameters used for comparison of thresholding


	**Global**	**Adaptive**	**Double adaptive**
	**thresholding**	**thresholding**	**thresholding**
	tv: 150	ms: -1.25	nh: 15
Default	cs: 4000	cs: 4000	cs: 4000
parameters	type: binary	bs: 19	bd: 5
		type: binary	
	tv: 149/159	ms: -2.50/-2.32	nh: 42/36
Optimized	cs: 15/0	cs: 1024/457	cs: 306/219
parameters	type: binary	bs: 200/27	bd: 5/5
		type: binary,mean_c	

In Figure 4, parameters denote tv = threshold value, cs: minimal connected component size, ms: mean shift, nh: neighborhood size, bs: block size, bd: bound drop value, type: threshold option. The manually optimized parameters correspond to the examples in Figure 4, in top-down order. Extended definitions of the meaning of these parameters are found in Table 2 and the Manual.

In the first example, annotation (1) shows improved root separation of adaptive thresholding (AT) in comparison to global thresholding (GT). Annotation (2) shows a tendency of double adaptive thresholding (DAT) to omit dense clusters of roots; in the given example this omission occurs in the upper root crown. The optimized parameter set shows additional crown roots (3) achieved with GT and even additional lateral roots (4) with AT. Although lateral roots are less resolved for DAT compared to AT, the root networks exhibit less noisy edges (5), which improves estimates of traits that rely on skeleton extraction.

In the second example, annotation (6) shows a major drawback of GT. The dense part of the root crown is not resolved into single roots. By comparison, AT provides a clear separation of the individual roots (7) and DAT shows the loss of root structure (8) for the default parameter set. The manually optimized parameters again show additional fine roots in the upper root crown and slightly better root separation (9). Annotations (10) and (11) show comparable results of AT and DAT. Both AT and DAT were able to resolve lateral roots. Although the default parameters may be suitable for many use cases, we recommend the modification of thresholding parameters to suit specific needs.

### Custom traits can be integrated into the GiA Roots pipeline

**Figure 5:**
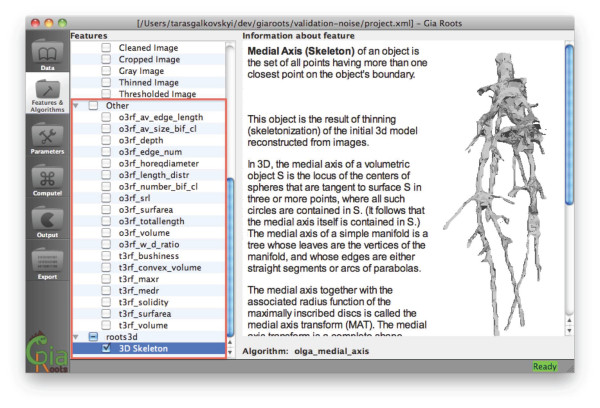
**Schematic of GiA Roots extended with new trait estimation algorithms.** A set of new traits is highlighted in the “Features & Algorithms” panel of GiA Roots. Installing new plugins is accomplished by copying the compiled plugin and documentation into the GiA Roots folder.

GiA Roots can be extended by copying compiled modules and accompanying information into the GiA Roots folder. Hence, GiA Roots does not require recompilation or any change of the software processing pipeline to include new traits or algorithms. In order to demonstrate this feature, we show how GiA Roots can be augmented to analyze 3D reconstructions of root networks. Although these features are still in development, we intend to release them in future versions of the software. As can be seen in Figure 5, a set of new traits was added to the software. The GUI of GiA Roots will automatically detect the new traits and a new checkbox will appear in the trait selection panel.

The extension of GiA Roots to include 3D trait estimation was accomplished as follows: (i) A new series of algorithms were written in C++ following the protocol for APIs (see Manual). (ii) The algorithm was compiled as a library. (iii) The compiled library was copied into the library directory of the GiA Roots directory structure. (iv) Visual depiction and textual description of the trait was added into the info directory including both an .html file description of the trait and a .png image file for display in the GUI – this step is not necessary, but it is helpful and encouraged. (v) GiA Roots was run, without recompilation, and the new trait functionality and its description are available to the user.

### Caveats and limitations

The objective of GiA Roots is to improve the high-throughput estimation of RSA traits from root network images. Hence, the caveats and limitations of the software reflect the design choices associated with meeting this objective. First, GiA Roots does not include the ability to interactively measure properties of individual networks (e.g., distance between two arbitrary points on the root network). Hence, if image quality varies significantly from image to image, then a more interactive software that allows greater user-interaction with images is likely to be more appropriate. Next, the quality of trait estimation in GiA Roots depends on the quality of segmentation. Our solution is to have the user select amongst thresholding algorithms with pre-defined parameter settings that we have found to be efficient in extracting root networks from background. These algorithms work whether roots are light on a dark background or vice-versa. However, parameters can also be changed by the user which are then store for full reproducibility of output. Care should be taken by users to validate the performance of these segmentation algorithms on their data. Finally, GiA Roots is extensible like some, but not all other software tools (see Table 4). However, the API is currently only available for libraries written in C++. Hence, developers require specific expertise in order to add new functionality.

**Table 4:** Comparison of root system analysis software


	**GiA Roots**	**EZ-Rhizo**	**SmartRoot**
License	free, closed-source	free, closed-source	free, closed-source
Platform	Mac, Windows	Windows only	cross-platform (an ImageJ plugin)
Language	C++	C++	Java
Root tracing	only automated	manual and automated	manual and semi-automated
Batch processing	built-in	no	no
Extensibility	C++ Plugin-API	none	none
Database support	no, but Excel compliant export	SQL	SQL

A feature comparison of GiA Roots with two free, recently released alternatives: EZ-Rhizo [24] and SmartRoot [39]. Note that traits available for estimation in GiA Roots are predominantly geometric, whereas EZ-Rhizo and SmartRoot estimate traits that are predominantly related to the connectivity of the root system. Hence, the traits estimated in each software do not necessarily coincide; more details can be found in the original papers.

### Future plans for development

GiA Roots is a general framework for analyzing the shapes of root systems. We anticipate extending GiA Roots to address problems that are direct extensions of the current software as well as explore possible applications to closely related problem domains.

First, a number of technological innovations now permit the 3D reconstruction of root systems. For example, an imaging platform and software tool for 3D reconstruction of rice root systems was recently introduced that utilizes multiple images of the same root system grown in a transparent gel [22]. As we have already shown, it is possible to extend GiA Roots to include traits that operate directly on 3D voxel data rather than sets of 2D images. We plan to build a robust set of trait estimation tools for 3D voxel data as part of a future version.

Second, root systems are dynamic, they grow heterogeneously in space and in time. Hence, we envision linking images of root systems (whether in 2D or 3D) in order to track the growth of the entire root system or components thereof. A similar approach has been espoused using point and click approaches [22], however we will attempt to fully automate the root tracking problem.

Third, we will explore how GiA Roots can be extended to analyze other plant phenomics problems. For example, a subset of us (CAP, YM, OS and JSW) collaborated on the development of LEAF GUI [44], a user-assisted software for the extraction of leaf venation networks. LEAF GUI enables users to utilize a set of transformations in a non-linear fashion to clean images prior to analysis. Hence, it is ideally suited for analyzing archival image data of varying quality. However, large-scale analysis of hundreds, thousands or more leaf images may benefit from matching the capabilities of interactive software like LEAF GUI with the more restrictive pipeline of GiA Roots. We note that other software is already available to characterize leaf shape [45], and so we emphasize that our efforts will focus on physical plant networks for which many of the tools of GiA Roots will apply.

## Conclusions

GiA Roots is a software framework for the high-throughput analysis of root system architecture. GiA Roots can analyze a single image up to many thousands of images, and for each image, extract the root network, estimate its traits, and report quantitative trait estimates and processing intermediates back to the end-user. GiA Roots is designed for end-users with limited technical background and for developers who wish to integrate a root trait estimation framework into their workflows. As such, GiA Roots is comprised of two tools: a GUI and a command line tool. The two tools perform all trait estimation steps using the same image processing pipeline. We anticipate that most users will utilize the GUI exclusively, whereas technical users will also utilize the command line tool. Importantly, GiA Roots is extensible unlike some root trait estimation packages currently available. Hence, developers can add on new algorithms and trait estimation steps using plugins and have these plugins interface with GiA Roots using documented APIs. This extensible step will help shorten the development time between identification of a possibly important root trait and its inclusion in processing pipelines.

The ability to link genotype to phenotype in the service of plant biotechnology will require the contributions of many groups and the utilization of many molecular, physiological and imaging techniques. We envision that GiA Roots can be utilized to help link genotypic with phenotypic information by improving the pipeline for extracting RSA traits. The trait algorithms currently bundled in GiA Roots are designed to quantify the RSA of monocot plants. However, in the future we anticipate adding modules that can characterize RSA for dicots, quantify temporal growth, and estimate traits from 3D reconstructions.

## Availability and requirements

**Project name:** GiA Roots

**Project home page:**http://www.giaroots.org

**Accessibility** Academic users are encouraged to includetheir contact information before downloading thesoftware. Commercial users should contact JSW(http://jsweitz@gatech.edu) for more information on obtainingthe software.

**Operating system(s):** Binary distributions available forMicrosoft Windows 7 and Apple Mac OS X 10.6.x.

**Programming language:** C++

Frameworks and libraries used:

· Qt framework (http://qt.nokia.com/products) - a cross-platform application and UI framework. Copyright (c) 2008-2010 Nokia Corporation and/or its subsidiaries. Licensed under LGPL v2.1.

· OpenCV (http://opencv.willowgarage.com/wiki/) - a library of programming functions for real time computer vision. BSD License.

· tinyxml (http://sourceforge.net/projects/tinyxml) - a simple, small, minimal, C++ XML parser. Licensed under zlib.

**License:** Academic use is governed by the followinglicense. For commercial licensing contacthttp://jsweitz@gatech.edu.

GiA Roots - “Software for the High Throughput Analysis of Plant Root System Images”. Copyright (C) 2010-2012 Georgia Tech Research Corporation and Duke University.By downloading and/or utilizing this Program, you agree to become bound by the terms and conditions of this license. If you do not agree with the terms and conditions setforth below, do not use this Program or any portion thereof in any form or manner.

This Program is licensed, not sold to you (“User”), by Georgia Tech Research Corporation (“GTRC”), owner of all rights and title to the code and accompanying documentation (“Program”) for use under the terms and conditions of this license. As such, GTRC reserves any and all rights not expressly granted to User under this license. With respect to the Program to which GTRC has exclusionary rights, GTRC hereby grants to User a nontransferable, non-exclusive license to use the Program for User’s own educational and non-commercial research purposes only. GTRC shall have the right to terminate this agreement and/or license without cause at any time.

User accepts the Program on an “as is” basis. GTRC makes no representation or warranty that the Program will be accessible or downloadable. GTRC makes no warranty that all errors can be or have been eliminated from the Program. GTRC shall not be responsible for losses of any kind resulting from the use of the Program and its accompanying document, and can in no way provide compensation for any losses sustained, including but not limited to any obligation, liability, right, claim or remedy for tort, or for any actual or alleged infringement of patents, copyrights, trade secrets, or similar rights of third parties, nor any business expense, machine downtime or damages caused User by any deficiency, defect or error in the Program or malfunction thereof, nor any incidental or consequential damages, however caused. GTRC disclaims all warranties, both express and implied respecting the use and operation of the Program and its accompanying documentation, including all implied warranties of merchantability and fitness for particular purpose and any implied warranty arising from course of performance, course of dealing or usage of trade. The User of the Program is expected to make the final evaluation of the Program’s usefulness in User’s own environment.

## Abbreviations

GiA Roots, General Image analysis of Roots; API, Application programming interface; RSA, Root system architecture.

## Competing interests

PNB is CEO and co-founder of GrassRoots Biotechnology, which uses the gel platform as part of its root phenotyping efforts. JH and CNT consulted for GrassRoots Biotechnology. The remaining authors declare that they have no competing interests.

## Authors’ contributions

TG was the primary developer and coder of GiA Roots, co-designed the GiA Roots software framework, and assisted in writing the manuscript. YM co-designed the GiA Roots framework. AB performed analysis of GiA Roots on data and assisted with software implementation. OS designed and developed algorithms. CAP assisted with algorithm design and GUI requirements. BM assisted with user-interface design and usability for both the GUI and command line interface. PZ assisted with the GUI. ASIP contributed root images and contributed to user-interface and algorithm design. CT contributed to user-interface and algorithm design. SF contributed to user-interface and algorithm design. JH contributed to algorithm design. PNB contributed root images and contributed to algorithm design. JSW developed the project idea, designed and developed algorithms, and contributed to software design. JSW wrote the manuscript with contributions from TG, YM, AB, OS, CAP, CT, ASIP, and PNB. All authors read and approved the final manuscript.

## Supplementary Material

Additional file 1 **Comparison of GiA Roots to previous benchmark.** Compilation of statistical correlation of the output of GiA Roots against a previous benchmark code written in Matlab [21]. The comparisons are for 16 different traits estimated from 2393 previously thresholded rice root images taken from 12 genotypes. All 2393 images are available at http://www.rootnet.biology.gatech.edu/data/PlantPhysData.zip. Click here for file
