# Supplementary material for: GiA Roots: software for the high throughput analysis of plant root system architecture
Source: BMC Plant Biol. 2012 Jul 26;12:116. doi: 10.1186/1471-2229-12-116 (PMC3444351; doi:10.1186/1471-2229-12-116)
Supplement: Additional file 1 — Comparison of GiA Roots to previous benchmark. Compilation of statistical correlation of the output of GiA Roots against a previous benchmark code written in Matlab [21]. The comparisons are for 16 different traits estimated from 2393 previously thresholded rice root images taken from 12 genotypes. All 2393 images are available at http://www.rootnet.biology.gatech.edu/data/PlantPhysData.zip. [file 1471-2229-12-116-S1.pdf]

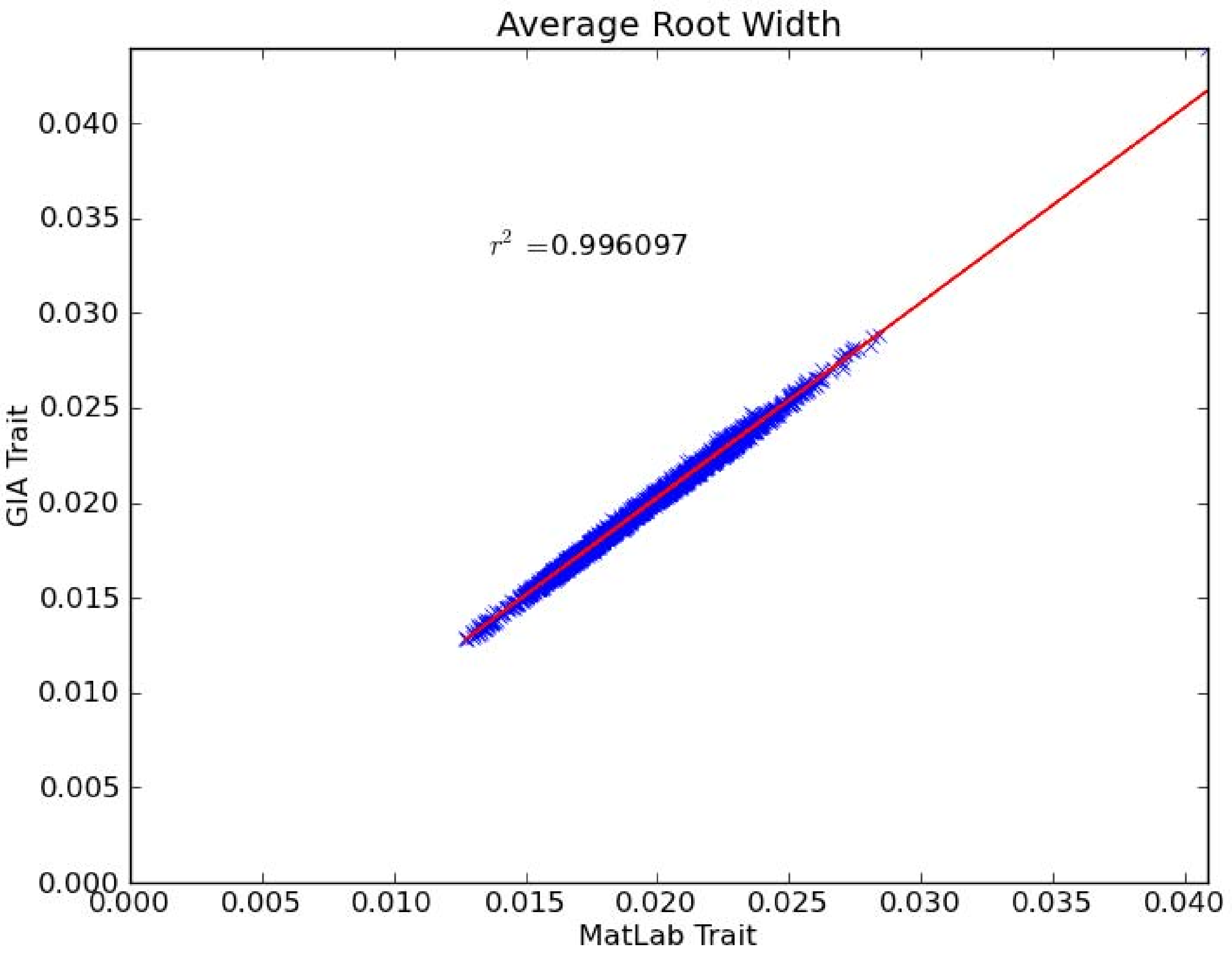

Average Root Width
r^2 =0.996097
GIA Trait
MatLab Trait
0.000
0.005
0.010
0.015
0.020
0.025
0.030
0.035
0.040

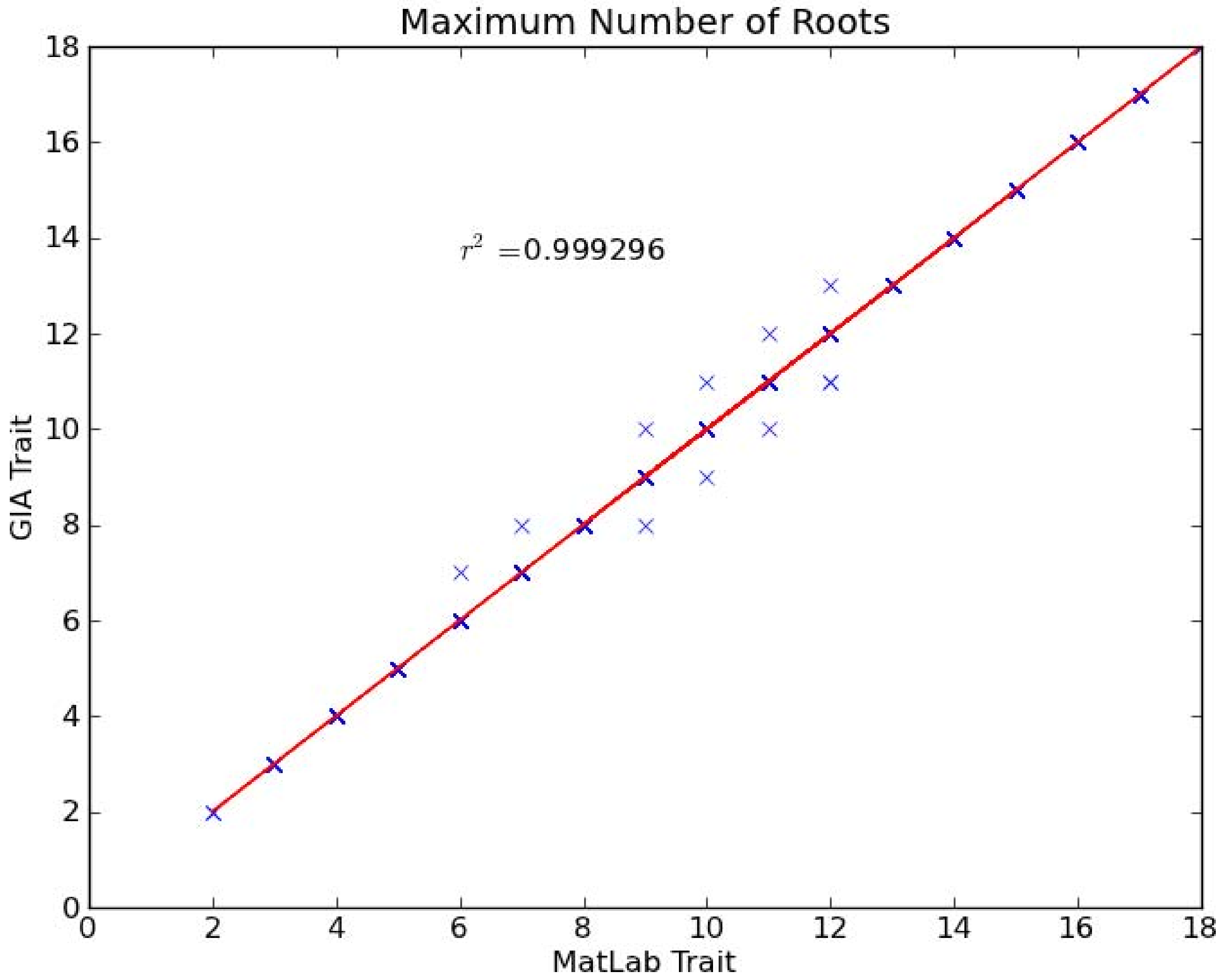

Maximum Number of Roots
r^2 =0.999296
GIA Trait
MatLab Trait
0
2
4
6
8
10
12
14
16
18

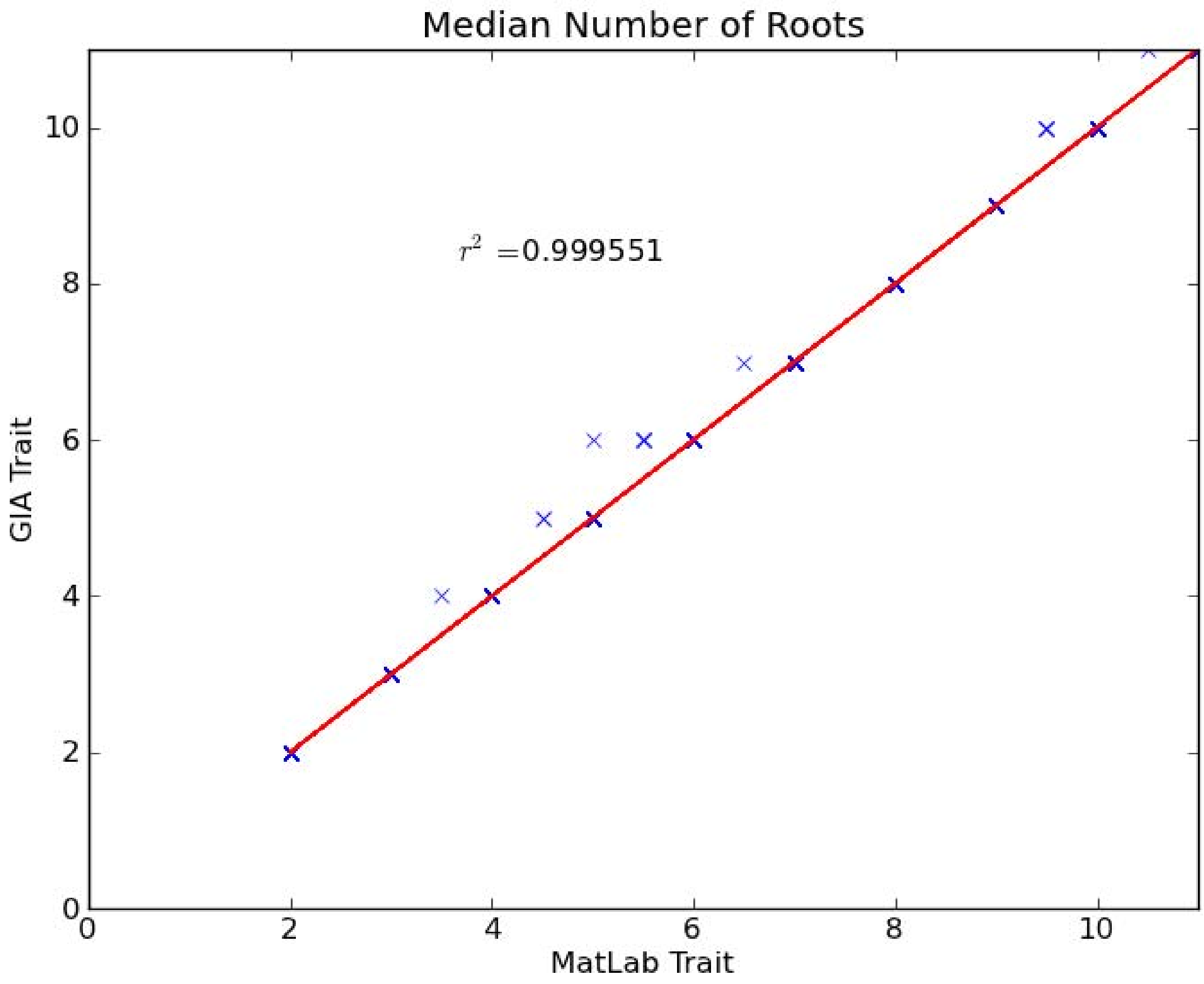

Median Number of Roots
r^2 =0.999551
GIA Trait
MatLab Trait
0
2
4
6
8
10

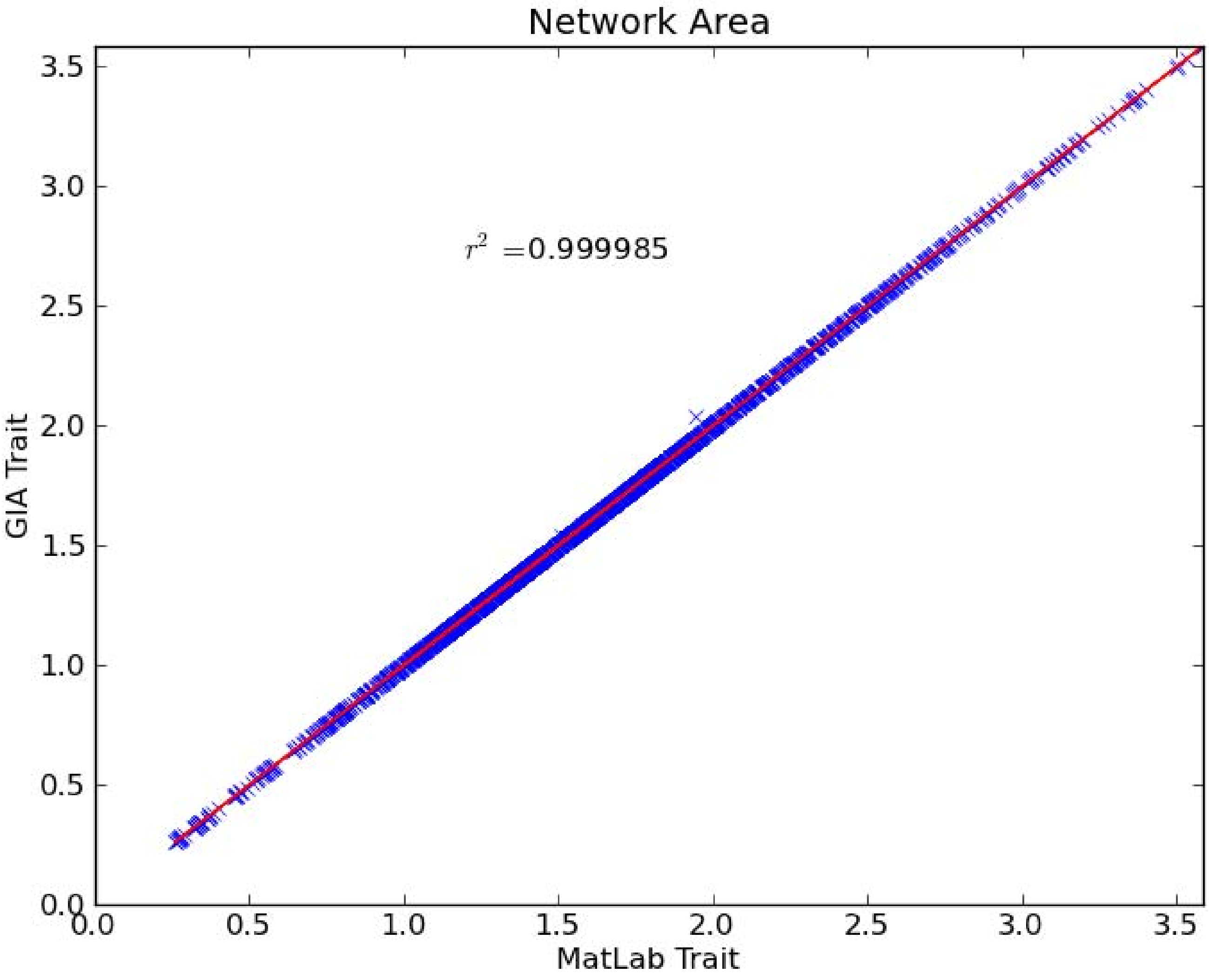
Network Area
r^2 =0.999985
GIA Trait
MatLab Trait
0.0
0.5
1.0
1.5
2.0
2.5
3.0
3.5

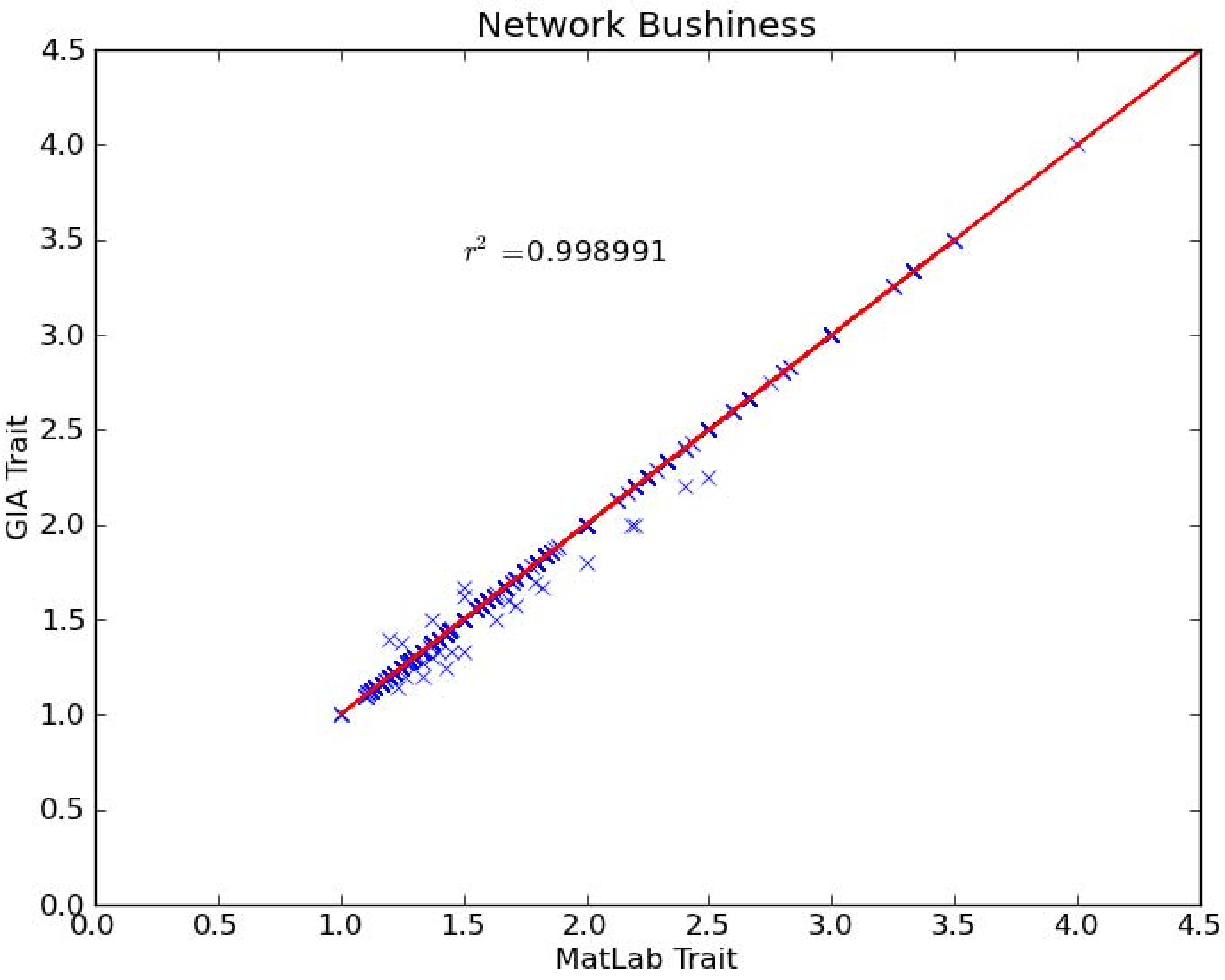

Network Bushiness
r^2 =0.998991
GIA Trait
MatLab Trait
0.0
0.5
1.0
1.5
2.0
2.5
3.0
3.5
4.0
4.5

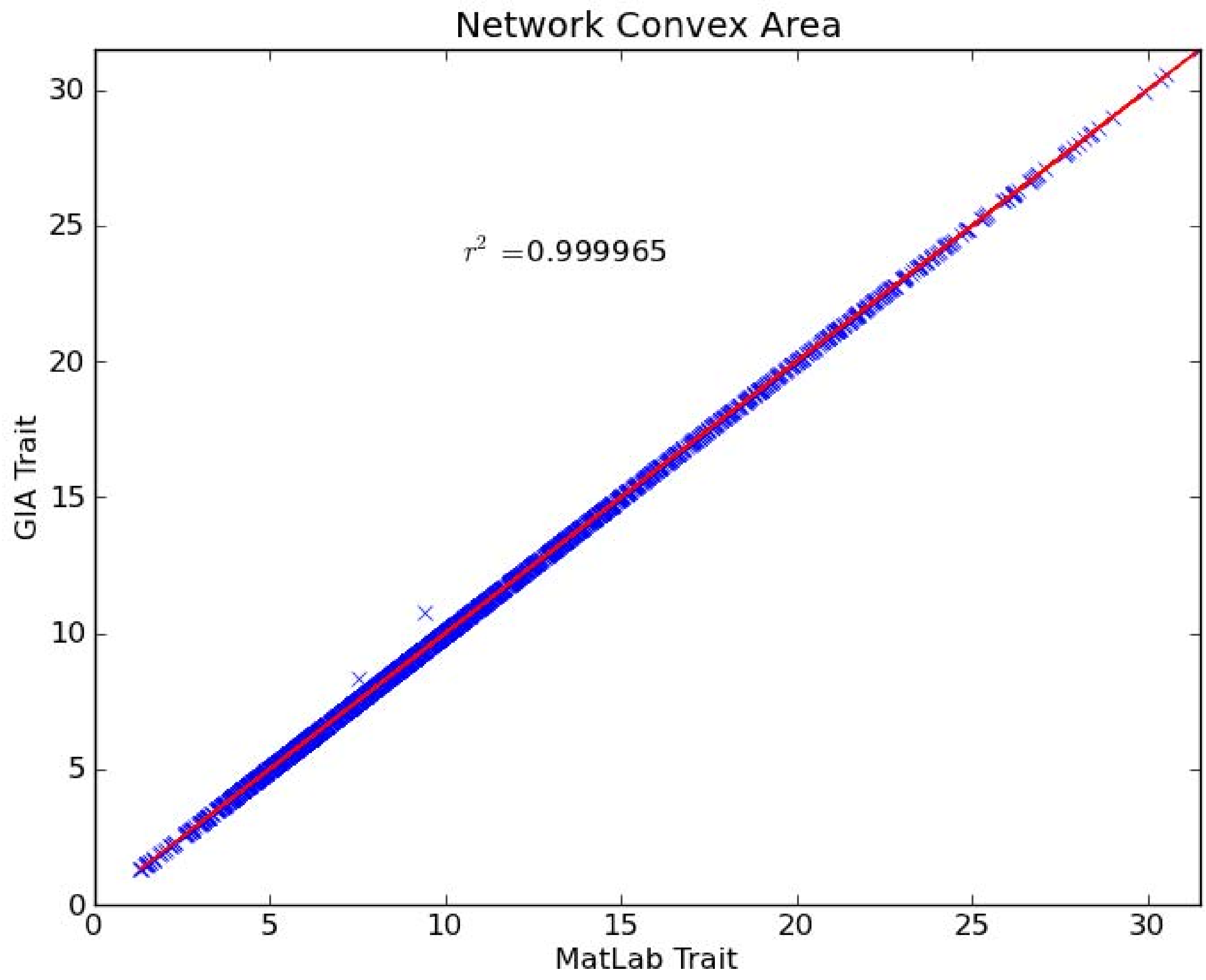
Network Convex Area
r^2 =0.999965
GIA Trait
MatLab Trait
0
5
10
15
20
25
30

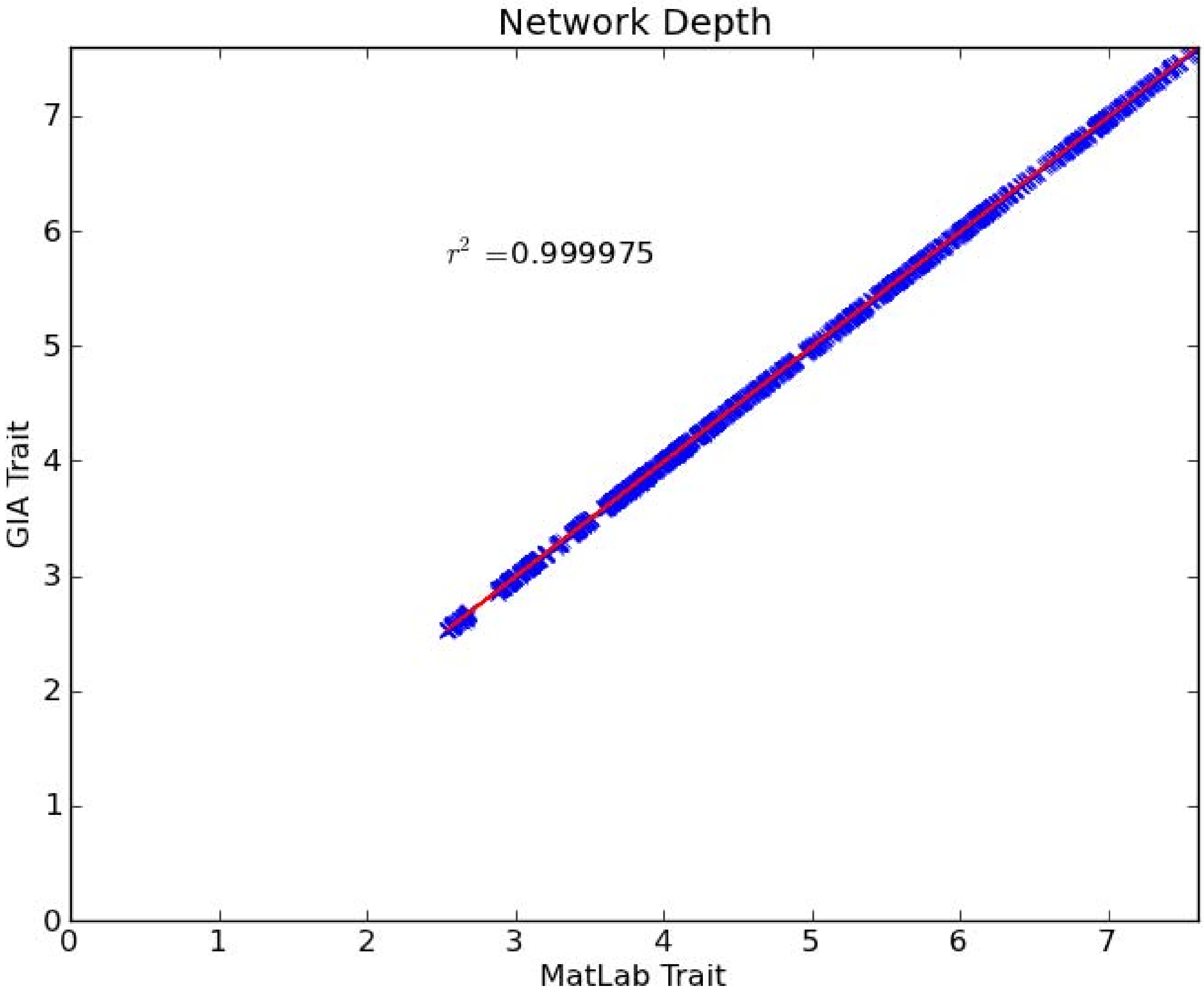
Network Depth
r^2 =0.999975
GIA Trait
MatLab Trait
0
1
2
3
4
5
6
7

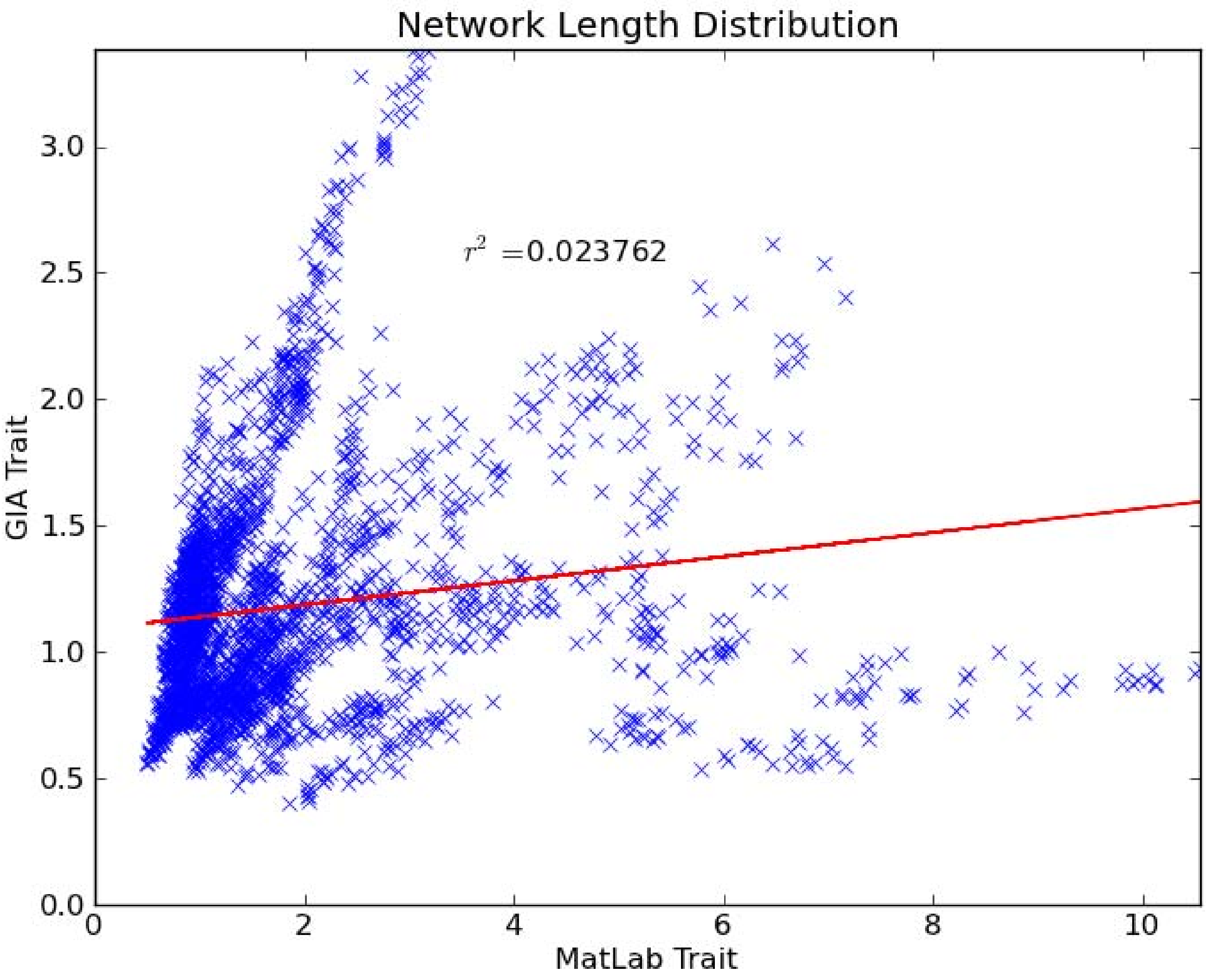
Network Length Distribution
r^2 =0.023762
GIA Trait
MatLab Trait
0.0
0.5
1.0
1.5
2.0
2.5
3.0
0
2
4
6
8
10

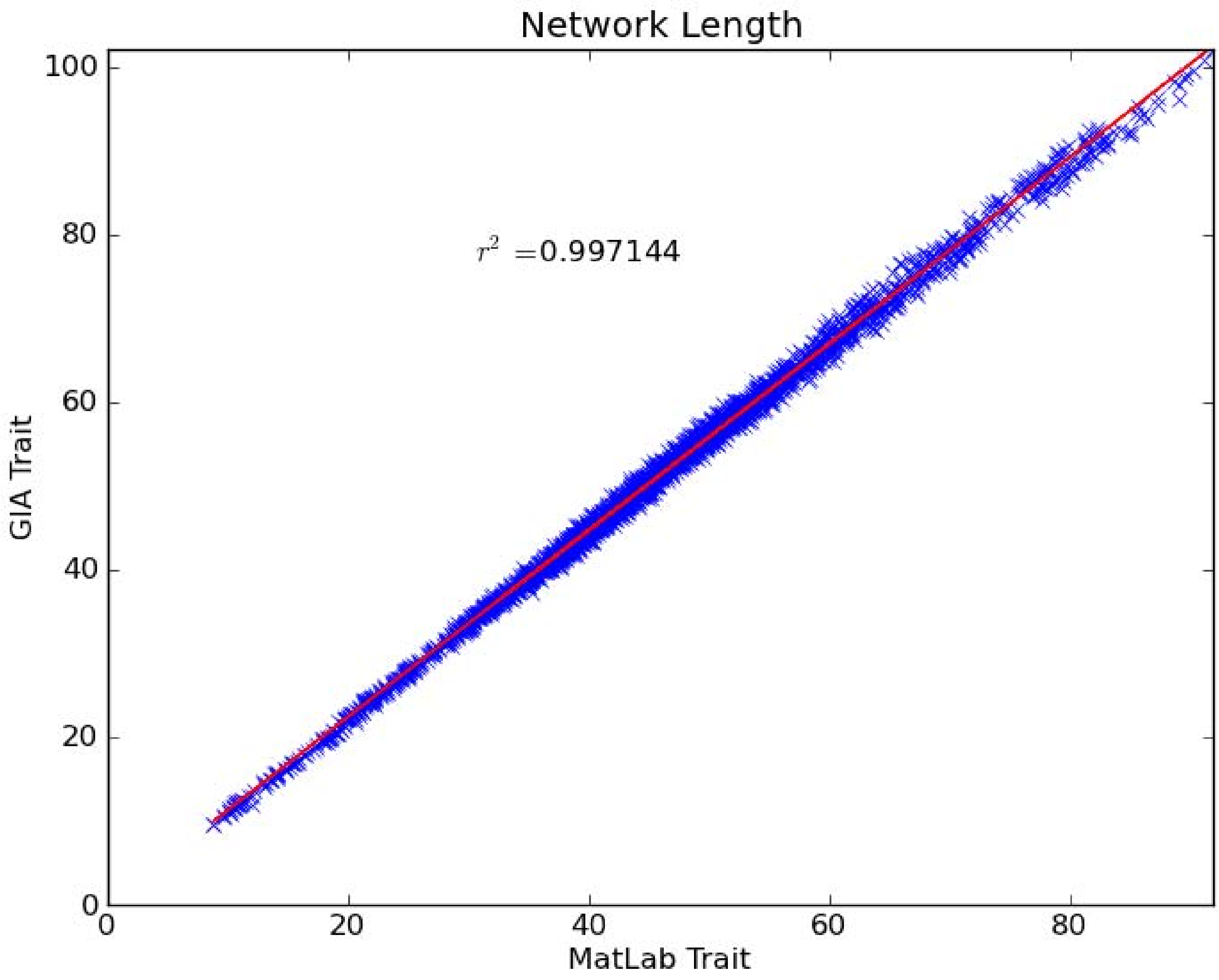

Network Length
r^2 =0.997144
GIA Trait
MatLab Trait
0
20
40
60
80
100
0
20
40
60
80

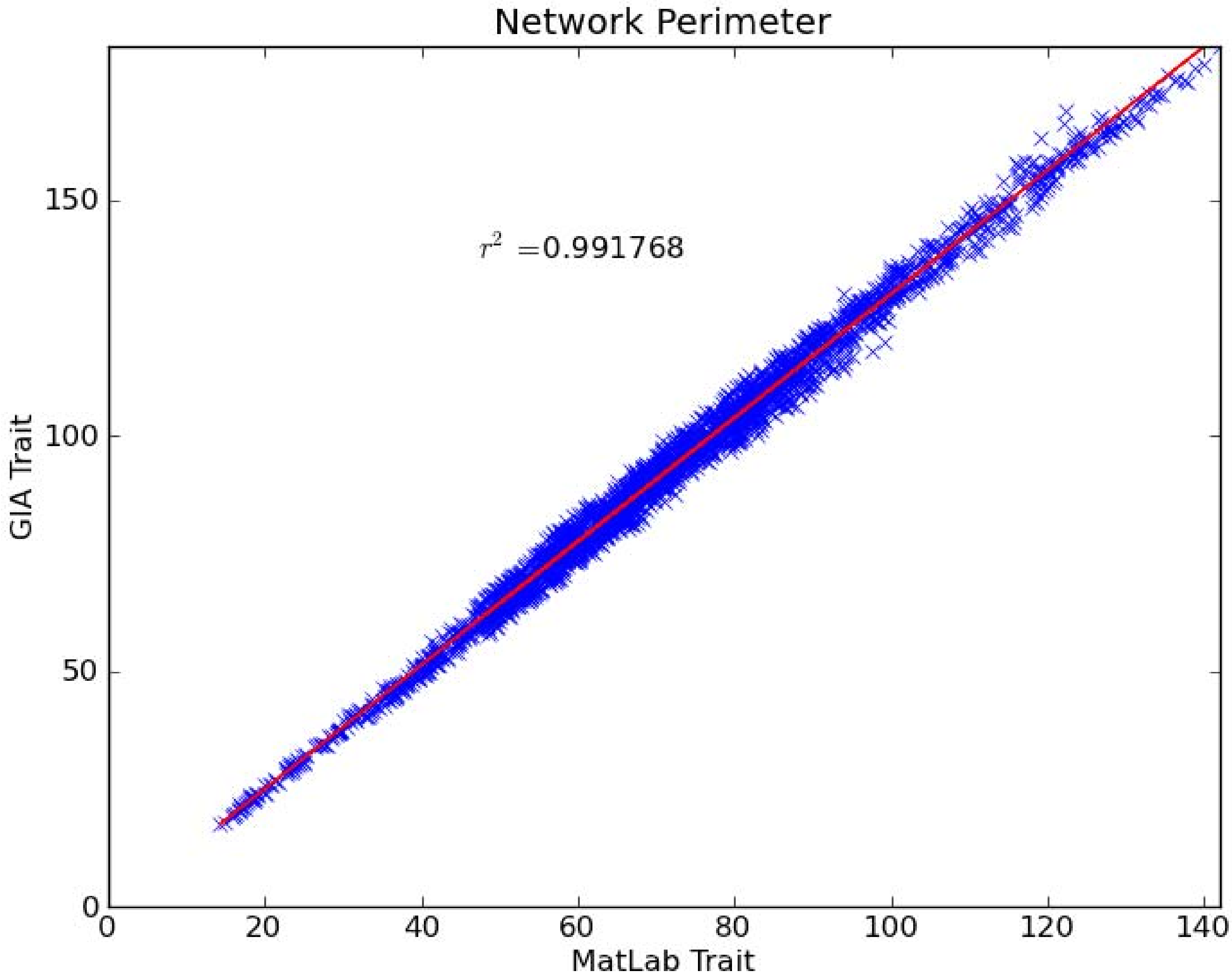

Network Perimeter
r^2 =0.991768
GIA Trait
MatLab Trait
0
50
100
150
0
20
40
60
80
100
120
140

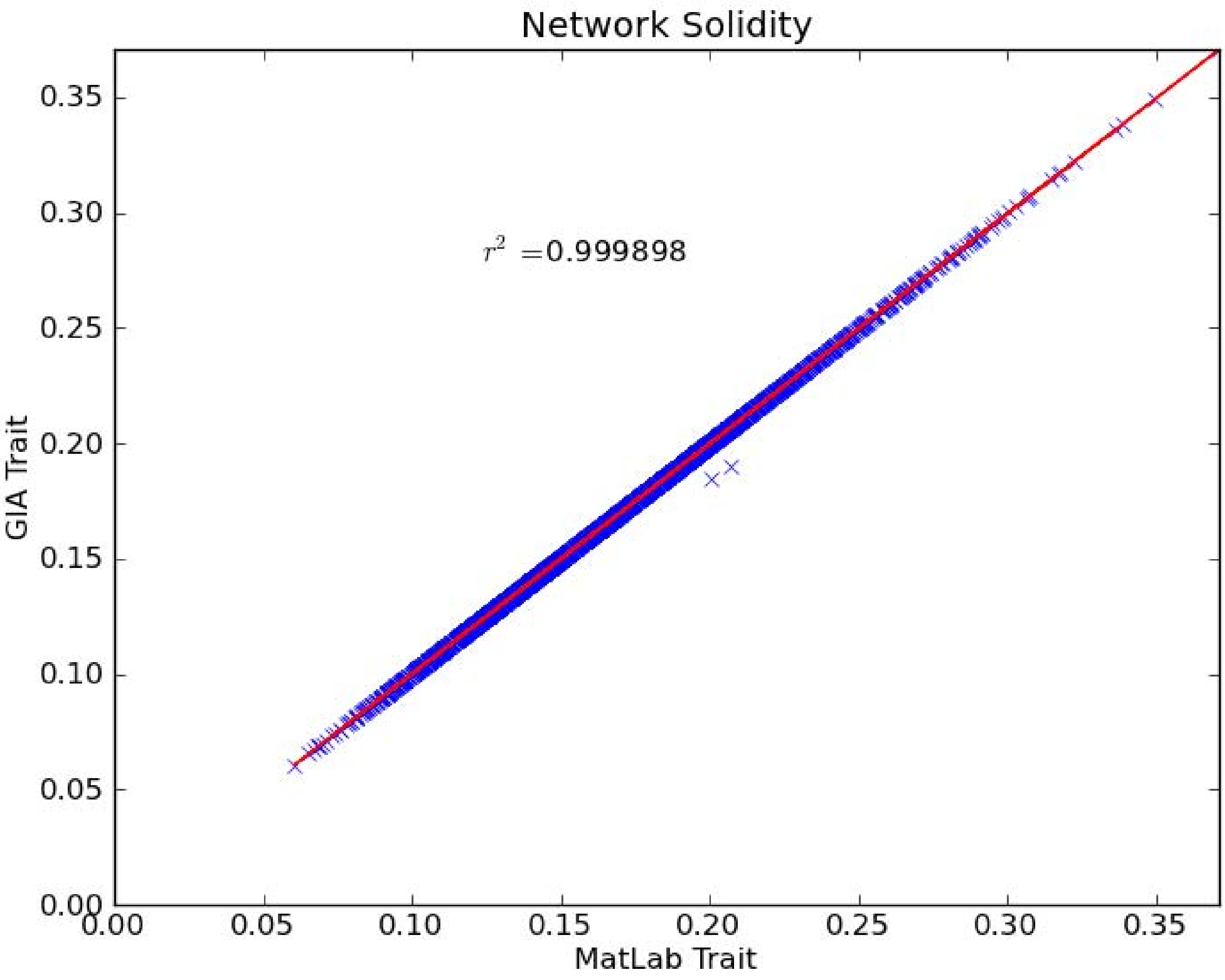
Network Solidity
r^2 =0.999898
GIA Trait
MatLab Trait
0.00
0.05
0.10
0.15
0.20
0.25
0.30
0.35

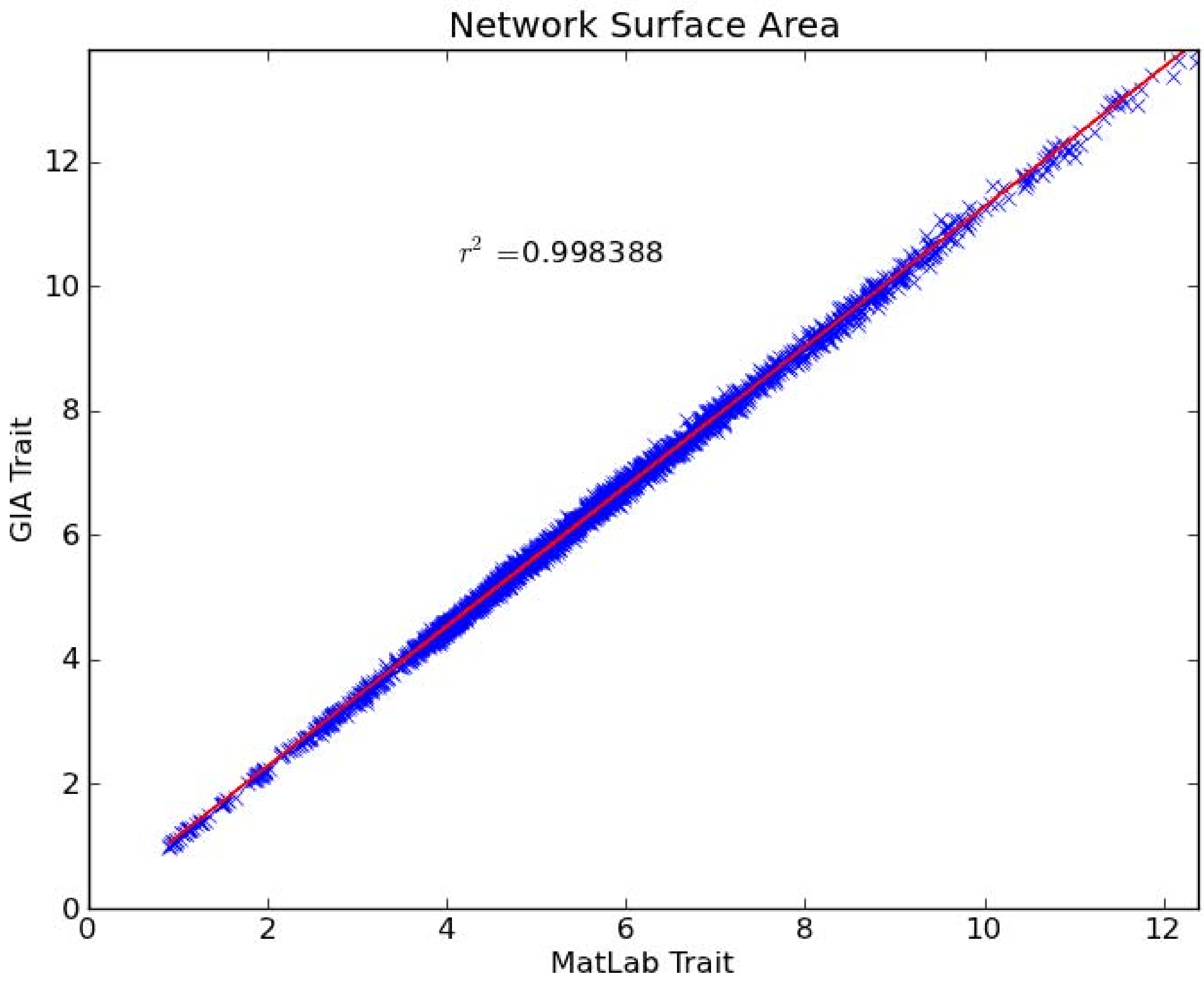

Network Surface Area
r^2 =0.998388
GIA Trait
MatLab Trait
0
2
4
6
8
10
12

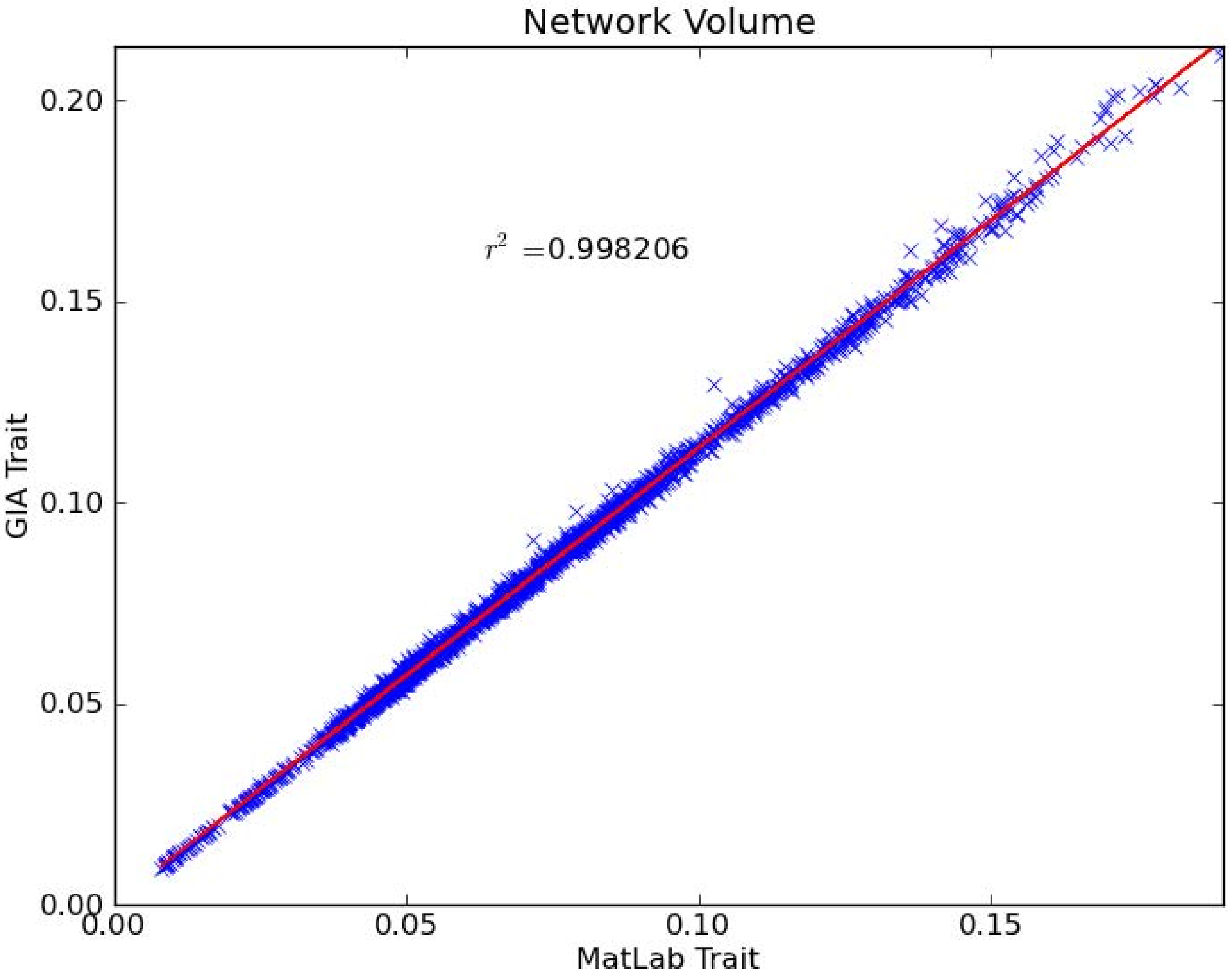
Network Volume
r^2 =0.998206
GIA Trait
MatLab Trait
0.00
0.05
0.10
0.15
0.20
0.00
0.05
0.10
0.15

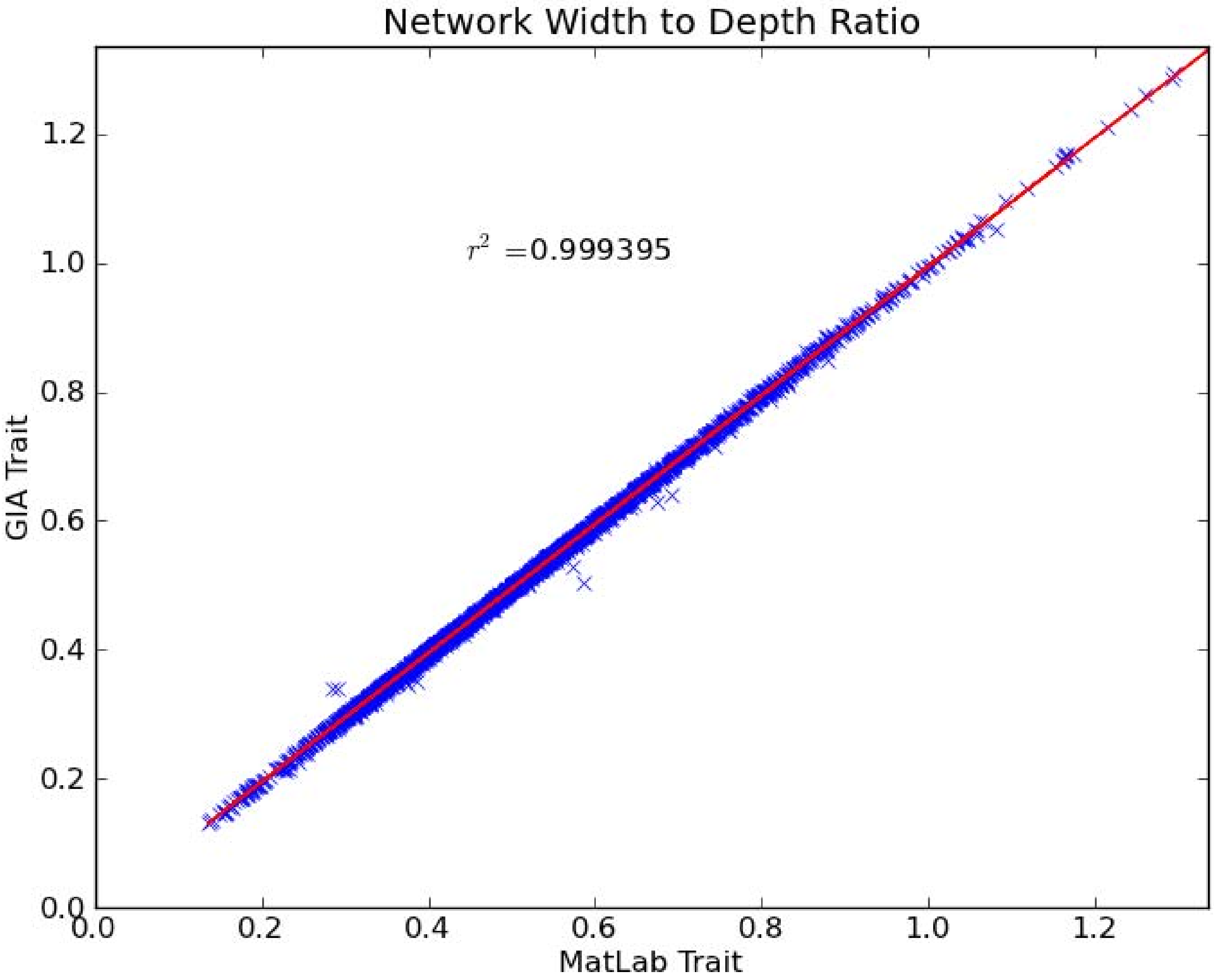

Network Width to Depth Ratio
r^2 =0.999395
GIA Trait
MatLab Trait
0.0
0.2
0.4
0.6
0.8
1.0
1.2

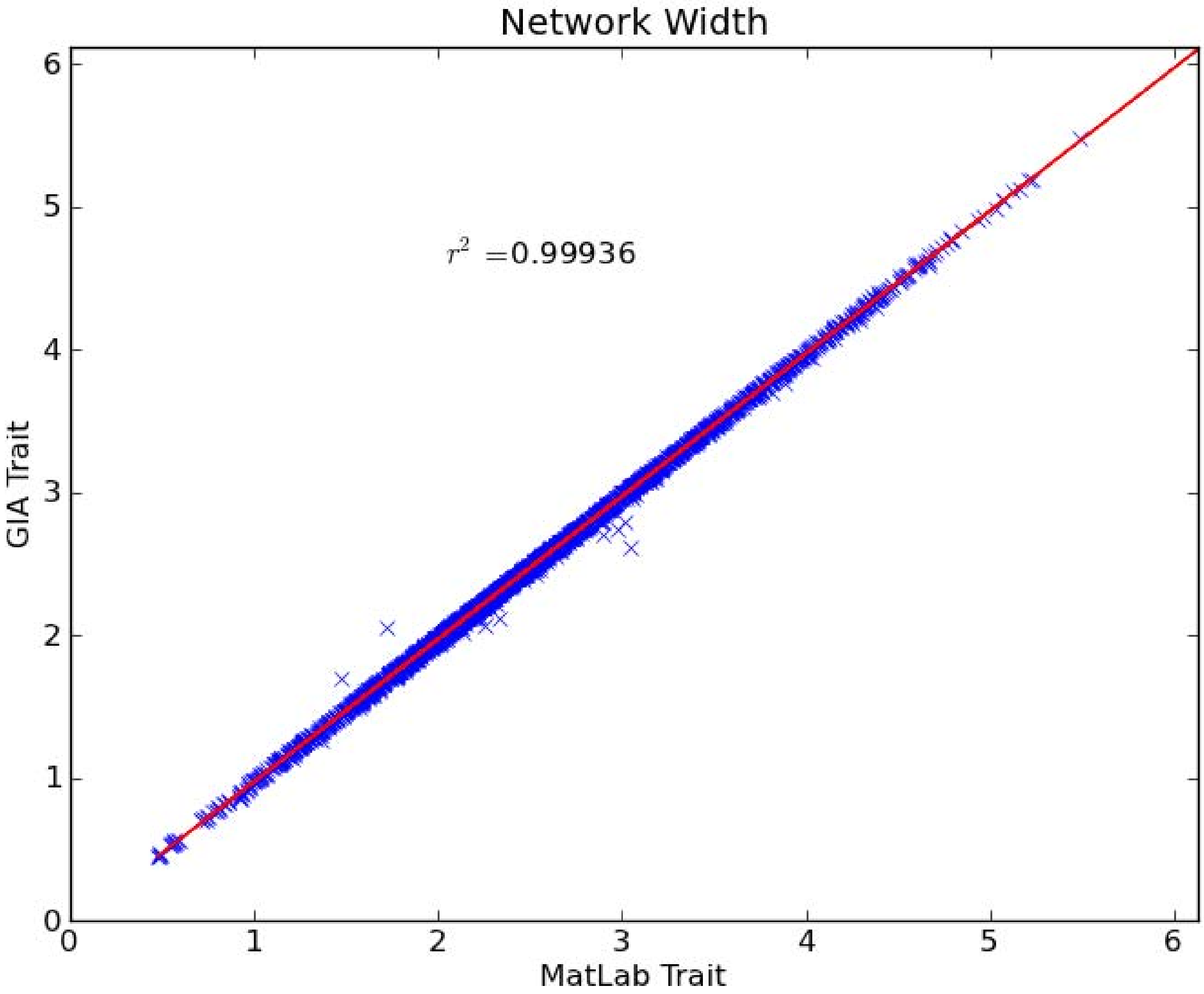
Network Width
r^2 =0.99936
GIA Trait
MatLab Trait
0
1
2
3
4
5
6

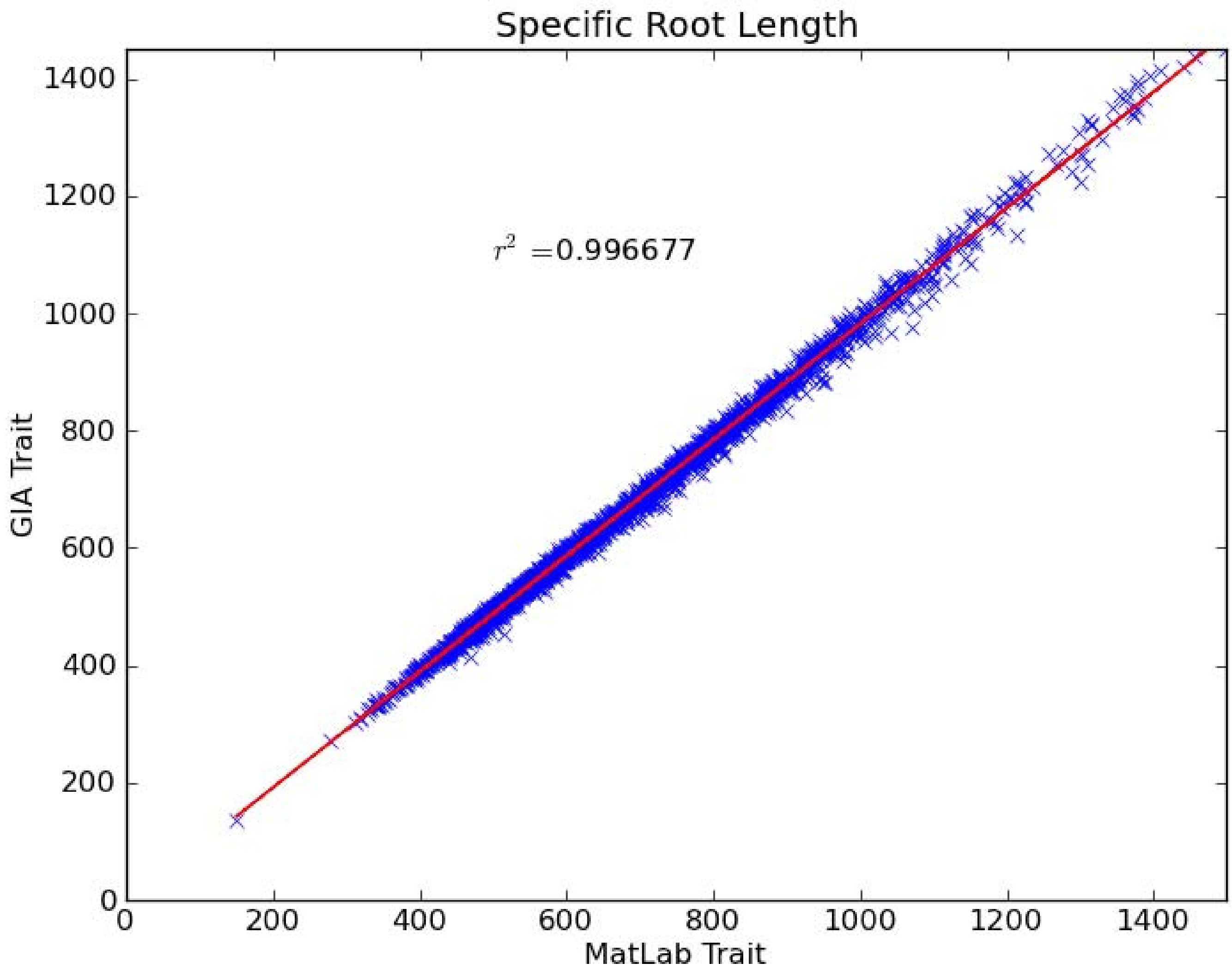
Specific Root Length
r^2 =0.996677
GIA Trait
MatLab Trait
0
200
400
600
800
1000
1200
1400
0
200
400
600
800
1000
1200
1400
